# Roles of Mitochondria in Oral Squamous Cell Carcinoma Therapy: Friend or Foe?

**DOI:** 10.3390/cancers14235723

**Published:** 2022-11-22

**Authors:** Junqiang Bai, Luping Wu, Xinmiao Wang, Yifan Wang, Zhengjun Shang, Erhui Jiang, Zhe Shao

**Affiliations:** 1 The State Key Laboratory Breeding Base of Basic Science of Stomatology (Hubei-MOST) & Key Laboratory of Oral Biomedicine Ministry of Education (KLOBM), School & Hospital of Stomatology, Wuhan University, Wuhan 430089, China; 2 Department of Oral and Maxillofacial-Head and Neck Oncology, School & Hospital of Stomatology, Wuhan University, Wuhan 430089, China

**Keywords:** OSCC, mitochondria, therapy, resistance, nanoparticles

## Abstract

**Simple Summary:**

Although many therapies have been applied in oral squamous cell carcinoma, these therapies are still unsatisfactory. Recently, the mitochondrial role in oral squamous cell carcinoma therapy has appeared, but related researches are limited and there are no related reviews from which to extract and condense such studies. Therefore, expatiating those studies is beneficial for us to comprehend the role of mitochondria in OSCC therapy and to derive a better outcome from it, even providing direction for other cancer therapies.

**Abstract:**

Oral squamous cell carcinoma (OSCC) therapy is unsatisfactory, and the prevalence of the disease is increasing. The role of mitochondria in OSCC therapy has recently attracted increasing attention, however, many mechanisms remain unclear. Therefore, we elaborate upon relative studies in this review to achieve a better therapeutic effect of OSCC treatment in the future. Interestingly, we found that mitochondria not only contribute to OSCC therapy but also promote resistance, and targeting the mitochondria of OSCC via nanoparticles is a promising way to treat OSCC.

## 1. Introduction

Oral squamous cell carcinoma (OSCC) usually occurs in the tongue, floor of the mouth, gingiva, palate and buccal mucosa and accounts for approximately 40% of head and neck squamous cell carcinomas (HNSCCs) [1,2]. Each year, approximately 300,000 new cases of OSCC are diagnosed worldwide, and the prevalence of OSCC has continued to increase [3,4]. Many therapies have been applied to OSCC [5]. However, the five-year overall survival rate of OSCC remains approximately 60% [6] due to therapy resistance and side effects [7,8]. Obviously, more effective therapies are needed for OSCC. 

The mitochondrion is the vital powerhouse of the cell [9]. Interestingly, additional functions of mitochondria other than energy production have been reported, including apoptosis induction, reactive oxygen species (ROS) production, mitochondrial fission and mitophagy [10]. Obviously, mitochondria play a significant role in the physiological and pathological processes in cells. Of note, increasing evidence shows that mitochondria have links to cancer [11]. 

In recent years, the relationship between mitochondria and OSCC, in terms of therapy, has attracted increasing attention. For example, casticin could induce apoptosis in OSCC by regulating the mitochondrial apoptosis pathway (MAP) [12]. Moreover, mitochondrial ROS (mtROS) can also induce cytochrome C (Cyt C) release by opening the mitochondrial permeability transition pore (mPTP), which causes the apoptosis of OSCC cells [13]. Interestingly, mitochondrial fission can also boost the production of mtROS and Cyt C [14,15]. However, other characteristics of mitochondria can cause therapeutic resistance [16,17]. For example, mitophagy could remove mitochondria damaged by ROS, thereby decreasing the effect of ROS-mediated therapy in OSCC [18]. In addition, abnormal nucleic acids in mitochondria can also interfere with therapy by regulating cellular metabolism to meet the need for cancer cell survival [19,20], and some constituents of the tumor environment (TME) interact with mitochondria in OSCC, which can also lead to resistance [21,22]. Regulating these mechanisms is obviously beneficial to OSCC therapy. Notably, nanomedicine, especially nanoparticles (NPs), has attracted substantial attention in this field for its accurate targeting of mitochondria [23].

**Figure 1:**
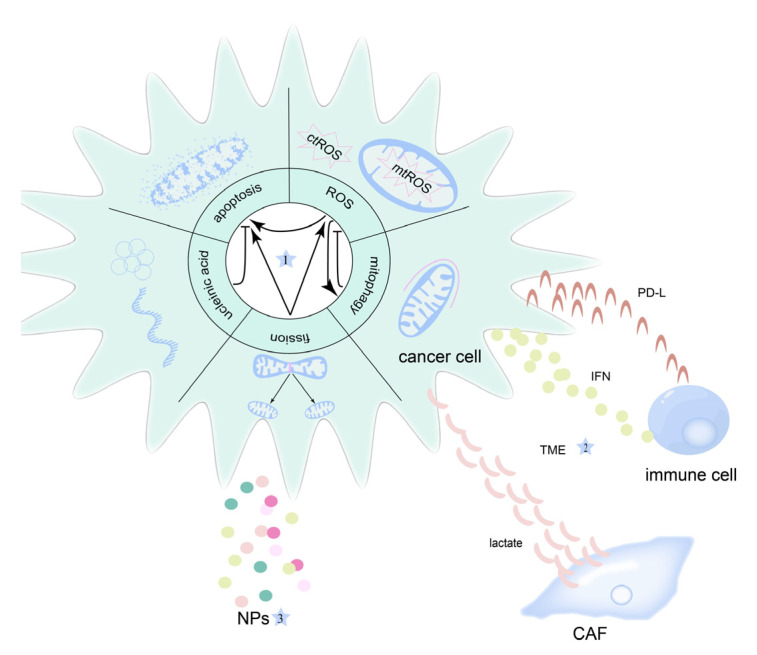
Overview of the role of mitochondria in the therapy and therapeutic resistance of oral squamous cell carcinoma (OSCC). (1) Mitochondrial function and their connection in OSCC. (2) Interaction between mitochondria and the tumor microenvironment (TME) constituents including immune cells and CAF (cancer-associated fibroblast). (3) Nanoparticles (NPs) were used to treat OSCC by targeting mitochondria.

In summary, the mechanisms that define the relationship between mitochondria and therapy or therapeutic resistance in OSCC are complex. Therefore, in this review, we expound on these mechanisms as completely as possible and briefly introduce the research progress of NPs that target OSCC via the above mechanisms (Figure 1).

## 2. Mitochondria and OSCC

Mitochondria, originating from symbiotic bacteria, accordingly have their own ~16.5 kb DNA (mtDNA), which can encode tRNAs, rRNAs and proteins vital for cell survival [24,25]. Physiologically, mitochondria play an important role in the bioenergetic, catabolic and anabolic processes [24,26,27,28]. Interestingly, mitochondria also play an important role in cancer biology. In 1924, Otto Warburg first found that cancer cells proceed with glycolysis to produce adenosine triphosphate (ATP) even in the presence of sufficient oxygen, which was termed the Warburg effect [29]. Further studies, however, found that the Warburg effect appeared in 70–80% of human cancers rather than all [30]. Nowadays, tumors with abnormal metabolism have arrived at a consensus [31], and mitochondria are the key point in this field [32]. Indeed, mitochondria have a tight link to cancer. First, mitochondria contribute to tumorigenesis via the accumulation of intermediate metabolites of the tricarboxylic acid cycle, activation of the nuclear factor erythroid 2-related factor 2 pathway, ROS, etc. [33]. Second, mitochondria are associated with cancer stem cells (CSCs) through mitochondrial fission, mitochondrial fusion and mitophagy [34]. Third, mitochondria promote metastasis by regulating motility and invasion, the TME, plasticity and colonization [35]. Undoubtedly, mitochondria participate in the therapeutic resistance of cancer [36]. Therefore, mitochondria, from another point, are regarded as a promising therapeutic target [37].

Considering the vital role of mitochondria in cancer biology, it is time to pay more attention to mitochondria in OSCC under the context that OSCC therapy is unsatisfactory. Indeed, OSCC has an abnormal metabolic pattern. In a retrospective study of 576 OSCC patients without weight loss, the progression-free survival and disease-specific survival were worse in obese patients compared with normal-weight patients, and 72 abnormal regulation lipid metabolism-related genes were identified in OSCC [38]. In addition, a systematic review with meta-analysis revealed that some components of energy metabolism might be the predictor of survival for the OSCC patient [39]. Although these studies did not reveal the direct role of mitochondria in these processes, considering the importance of mitochondria in metabolism, mitochondria, undoubtedly also play a multitude of roles in OSCC. For example, mitochondrial calcium uniporter was strongly expressed in OSCC compared with normal tissues, and the downregulation of mitochondrial calcium uniporter expressly impaired the proliferation and migration of OSCC cells [40]. The oxidation of nicotinamide adenine dinucleotide (NADH), a lactate derivative from cancer-associated fibroblasts (CAFs), in the mitochondrial oxidative phosphorylation (OXPHOS) of OSCC cells could promote the proliferation of OSCC [41]. Moreover, the M2 isoform of pyruvate kinase (PKM2), a regulator of mitochondrial functions [42], was strongly correlated with OSCC tumor progression [43]. 

The above studies show that mitochondria may be used as a therapeutic target for OSCC, therefore, we summarized the relevant studies on mitochondria in OSCC from the perspective of treatment. Intriguingly, we found that mitochondria contribute to both treatment and the resistance of OSCC.

## 3. Mitochondrial-Targeted Therapeutic Strategies for OSCC

Targeting the physiological process regulated by mitochondria, including OXPHOS [44], mitochondrial metabolism [45], mitophagy [46], mitochondrial fission and fusion [28] and apoptosis [47], is beneficial for cancer treatment. However, we found that regulating MAP, ROS and mitochondrial fission are the main ways to support OSCC treatment. This difference may due to the different occurrence sites. Anyway, illustrating the detailed mechanism of relative studies is worthy and indispensable for a better outcome of OSCC treatment.

### 3.1. Apoptosis

The harmony of Yin and Yang and the exchange of life and death are natural phenomena. Interestingly, mitochondria have this feature in cancer. On one hand, the energy produced by mitochondria is essential for cell survival, while on the other hand, mitochondria are instrumental in regulating cell deaths [48], including ferroptosis [49], cuproptosis [50], necroptosis [51], pyroptosis [52], and apoptosis [53]. Among these, stimulating apoptosis is a satisfactory means to treat malignant cells, and several clinical trials have demonstrated the feasibility of this approach [54,55].

Apoptosis can be induced by extrinsic or intrinsic pathways [56], while mitochondria mainly mediate the intracellular apoptosis pathway known as intrinsic apoptosis or MAP [57]. The Bcl-2 (B-cell lymphoma 2) protein family is primarily responsible for MAP [57]. This family can be divided into three subgroups: antiapoptotic proteins (such as Bcl-2, Bcl-xl, Mcl-1), proapoptotic proteins (Bax, Bak, Bok) and BH3-only proteins (Bad, Bid, Bim, etc.) [58]. Pores in the mitochondrial outer membrane can be induced by activated Bax or Bak, which results in mitochondrial outer membrane permeabilization (MOMP) and subsequent cytochrome C (Cyt C) release from mitochondria [59,60]. Along with apoptotic peptidase activating Factor 1, Cyt C forms the apoptosome, which activates caspase-9 and in turn activates caspase-3 and caspase-7, leading to apoptosis [61,62]. Obviously, regulating Bcl-2 protein family members is the key process to inducing MAP. 

Three dominating pathways could induce MAP by regulating the Bcl-2 family in SOCC. The first is the c-Jun N-terminal kinase (JNK) signaling pathway [63]. For example, phosphorylated JNK1/2 mediated the phosphorylation of Bak and Bim to reduce Bcl-2 and Bcl-xl expression; consequently, MAP occurred in tongue squamous cell carcinoma (TSCC) cells [64]. In addition, phosphorylated JNK1/2 could activate the ERK1/2/GSK3-α/β pathway to decrease the expression of Bcl-2 and to upregulate Bax, caspase-3 and caspase-7 in TSCC cells [65]. Second, the protein kinase B (AKT) signaling pathway is related to MAP. For example, enhanced JNK1/2 activation with decreased AKT activation could induce extrinsic apoptosis and in turn activate MAP in OSCC [66]. Therefore, inhibiting the phosphorylation of AKT dephosphorylated Bad, and led to the decreased expression of Bcl-2 and Bcl-xl and the subsequent activation of caspase-9 and caspase-3 in OSCC [67]. In addition, restraining the AKT/mammalian target of the rapamycin (mTOR) pathway could increase Bad expression and result in apoptosis of HNSCC cells [68]. Third, inhibited Janus kinase 2/signal transducer and activator of transcription 3 (JAK2/STAT3) signaling led to the expression of the proapoptotic proteins and the release of Cyt C, while the levels of Bcl-2, Bcl-xl and Mcl-1 were reduced, and thus resulting in MAP in OSCC [69]. 

In addition, regulating other cellular activities can also affect MAP in OSCC. First, MAP interacts with the extrinsic apoptosis pathway [70] because activation of the JNK pathway could increase the expression of caspase-8, which is a component of the extrinsic pathway, and could upregulate t-Bid and Bak in OSCC [66]. Indeed, the activation of extrinsic apoptosis promoted t-Bid expression, which activated Bak or Bax [48,71], resulting in MAP. Second, pyroptosis also has a relationship with MAP. Pyroptosis of head and neck cancer cells was regulated by Bax–Bad–caspase-3 because the inhibition of Bax and Bad could weaken the activation of gasdermin E, thereby preventing gasdermin E-mediated pyroptosis [72]. Finally, MAP is related to epigenetic regulation because the inhibition of the enhancer of Zeste homolog 2 in OSCC may increase intracellular ROS and result in an increased proportion of Bax/Bcl-2 and Cyt C release [73]. 

**Figure 2:**
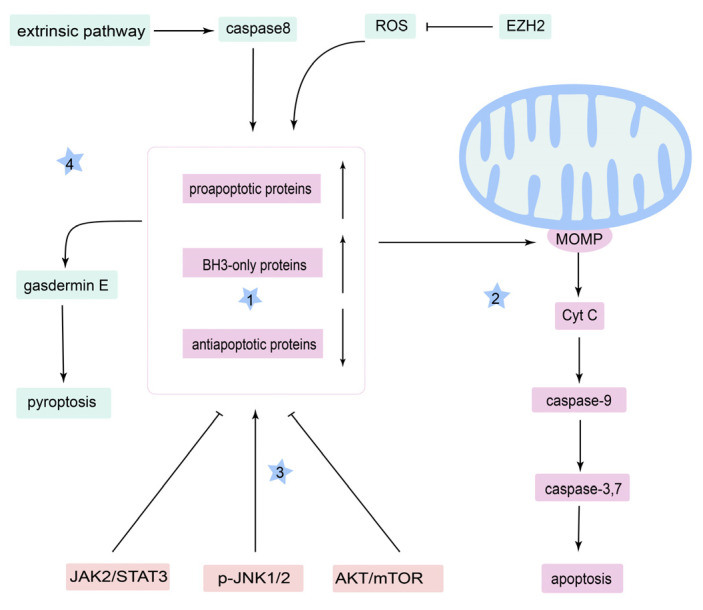
Mitochondrial apoptosis pathway in OSCC therapy. (1) Upregulation of the BH3-only proteins and the proapoptotic proteins, and reduced expression of the antiapoptotic proteins can cause (2) mitochondrial outer membrane permeabilization (MOMP) to promote the release of cytochrome C (Cyt C), which involves the activation of caspase-9 and in turn activates caspase-3 and caspase-7, leading to apoptosis. (3) Signaling pathways regulate the B-cell lymphoma 2 (Bcl-2) protein family to induce MOMP. (4) Other cell activities related to the mitochondrial apoptosis pathway.

Although these studies reveal that inducing mitochondria-relative cell deaths, especially apoptosis, is a rational therapy of OSCC (Figure 2), whether and how mitochondria explicitly regulate other cell death mechanisms besides apoptosis, such as cuproptosis, remains unclear in OSCC.

### 3.2. Reactive Oxygen Species

ROS play various roles in the biological processes of both normal and cancer cells [74]. ROS are mainly derived from the mitochondrial electron transport chain (ETC), which is composed of five protein complexes (complexes I–V), among which complexes I and III are the primary sites of ROS production [75,76]. Moreover, phagosomes, the endoplasmic reticulum (ER) and cellular membranes can also produce ROS [77]. Therefore, ROS can be divided into two groups: cytoplasmic ROS (ctROS), which have nonmitochondrial origins, and mitochondrial ROS (mtROS), which originate in mitochondria. Moderate levels of ROS promote cancer cell proliferation, while high levels of ROS cause cell death by damaging proteins, nucleic acids, lipids and other cellular components [78,79]. Therefore, improving ROS levels is a rational way to treat OSCC. 

Upregulating mtROS can cause MAP in OSCC. This is because excessive mtROS leads to the opening of the mitochondrial permeability transition pore (mPTP) and in turn, releases proapoptotic factors, including Cyt C, from the mitochondria [80,81,82,83] thereby causing MAP [61]. For example, azoxystrobin, an inhibitor of complex III, could suppress cell proliferation and cause MAP of OSCC cells by promoting mtROS formation [13]. In addition, antimycin A, another inhibitor of complex III, could also lead to the MAP of OSCC cells by inducing the production of mtROS [84]. Interestingly, ROS can influence Bcl-2 family proteins by regulating their phosphorylation and ubiquitination through many signaling pathways [85,86]. For example, ctROS could activate the JNK signaling pathway to upregulate Bax in OSCC cells [87]. Therefore, we can conclude that MAP induced by ROS occurs through (1) ROS destroying mitochondria, such as by opening the mPTP, to release Cyt C, and (2) ROS regulating Bcl-2 family proteins to cause MOMP.

Moreover, mtROS also play a therapeutic role by interacting with other molecules after diffusing from the mitochondria into the cytosol by free diffusion or by voltage-dependent anion channels [88,89]. First, mtROS can influence many signaling pathways. For example, mtROS could activate JNK, which induces caspase activation and apoptosis in OSCC [90]; mtROS could also activate the AMP-activated protein kinase (AMPK) pathway and inhibit the STAT3 pathway, which results in the suppression of the growth and proliferation of OSCC cells [91]. Second, mtROS can damage DNA, causing cell death [74,92]. As an example, usnea barbata could induce ROS, including mtROS, which induces the expression of γH2AX and 8-oxo-2′deoxyguanosine; these events contribute to OSCC cell death [93]. Similarly, manoalide can cause the overexpression of cleaved caspase-3, γH2AX and 8-oxo-2′deoxyguanosine, which leads to antiproliferative effects in OSCC resulting from oxidative stress, including mtROS [94]. Moreover, DNA damage and apoptosis induced by improved mtROS were also observed in the CSCs of OSCC [95].

Interestingly, these mtROS in the cytoplasm can also induce the production of additional mtROS by triggering the opening of the mPTP or the inner membrane anion channel, which in turn causes the synchronous breakdown of mitochondrial membrane potential (MMP) and a momentary increase in ROS production by the ETC [96]. This phenomenon is called ROS-induced ROS release [96]. Undoubtedly, ctROS also has this function. For example, ctROS damaged the MMP and induced the production of mtROS, which activated JNK and led to caspase activation in OSCC [90]. In addition, ctROS could cause the mPTP to open, after which MAP occurred in OSCC [97].

In addition to interacting with other molecules, ROS also serves as a bridge between the ER and mitochondria [98]. Specifically, ROS could cause ER stress, which results in the release of Ca^2+^; this Ca^2+^ was absorbed by mitochondria and caused excessive mitochondrial Ca^2+^ uptake, which opened the mPTP and released Cyt C [99,100]. Due to the opening of the mPTP, mtROS were upregulated [96,101]. Therefore, this mechanism can be used to treat OSCC. For example, enhancing the generation of ctROS could cause ER stress, which induced Ca^2+^ release and resulted in decreased MMP and subsequent apoptosis of OSCC cells [102]. Another study showed that Ca^2+^ from the ER could lead to the production of mtROS and the eventual apoptosis of OSCC cells [103]. 

**Figure 3:**
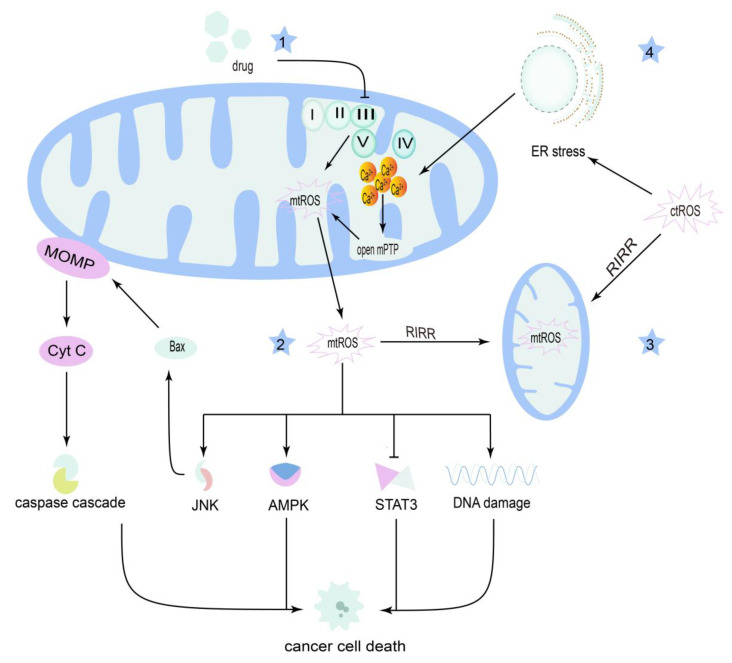
Reactive oxygen species (ROS) in OSCC therapy. (1) Drugs can induce the upregulation of mitochondrial reactive oxygen species (mtROS) by inhibiting the electron transport chain (ETC). (2) Those mtROS can suppress cancer cell survival by activating the c-Jun N-terminal kinase (JNK) and AMP-activated protein kinase (AMPK) pathways while inhibiting the signal transducer and activator of the transcription 3 (STAT3) pathway, causing DNA damage. (3) MtROS can be induced by mtROS and cytoplasmic reactive oxygen species (ctROS) via ROS-induced ROS release (RIRR). (4) CtROS can cause endoplasmic reticulum (ER) stress, resulting in a surge of Ca^2+^ in the mitochondria, and subsequently increased mtROS induced by the open of mitochondrial permeability transition pore (mPTP).

Therefore, ROS, including mtROS and ctROS, exhibit great potential as therapeutic targets for OSCC by affecting mitochondria (Figure 3). 

### 3.3. Mitochondrial Fission

Mitochondria are highly dynamically regulated by fission and fusion to cope with various cellular stimuli [104]. Mitochondrial fission is when one mitochondrion divides into two daughter mitochondria, while fusion occurs when two mitochondria combine into one mitochondrion [105]. Although mitochondrial fission is closely related to mitochondrial fusion, most of the recent studies on OSCC focused on mitochondrial fission.

Mitochondrial fission is regulated by many factors including dynamin-related protein 1 (Drp1) which can be transferred from the cytoplasm to the mitochondrial outer membrane, where it combines with its receptors, including mitochondrial fission factor [106] and mitochondrial fission protein 1 [107], to divide the mitochondria through constriction [108,109]. Various studies have found that mitochondrial fission supported the survival of cancer cells [110,111,112]. For example, mitochondrial fission could lead to the generation of new mitochondria and an increased total level of ATP, resulting in the high invasiveness of OSCC [113]. In addition, enhanced mitochondrial fission provided the daughter mitochondria that were needed for the rapid proliferation of OSCC cells [114], but impaired mitochondrial fission was associated with cisplatin resistance in OSCC cells [115]. Clearly, regulating mitochondrial fission is a therapeutic strategy for OSCC.

Suppressing mitochondrial fission can promote the apoptosis of OSCC cells. For example, cell viability and the ability of highly invasive OSCC cells to invade were suppressed by a naturally occurring quinone that was a substrate of reduced NADH phosphate dehydrogenase quinone 1, due to the inhibition of mitochondrial fission [116]. In addition, the decreased expression of phosphorylated Drp1 could delay the growth of cetuximab-resistant HNSCC patient-derived xenografts tumors [117]. Interestingly, inhibiting mitochondrial fission could facilitate immunotherapy in TSCC. Specifically, on one hand, reduced mitochondrial fission directly increased the membrane expression of major histocompatibility complex-I in TSCC; on the other hand, reduced mitochondrial fission decreased the production of ROS, which impaired the unfolded protein response of the ER and caused the subsequent suppression of the IRE1α–XBP-1s–TPP2 axis, resulting in increased levels of major histocompatibility complex-I [14]. 

Paradoxically, enhanced mitochondrial fission can also induce tumor cell death. For example, hypoxia-inducible factor 1α could directly bind to mitochondrial fission factor and increase its transcription, leading to increased mitochondrial fission and ultimately apoptosis by the induction of Cyt C release in OSCC cells [118], and increased Drp1 and fission protein 1, induced by the activated Wnt/β-catenin pathway, could also promote CAL-27 cell apoptosis by promoting Cyt C production [119]. Interestingly, altering the expression of long noncoding RNAs (lncRNAs) could enhance mitochondrial fission. For instance, lncRNA MPRL could induce sensitivity to cisplatin in OSCC. Mechanistically, MPRL decreased miR-483-5p expression by directly binding to premiR-483-5p and in turn increased fission protein 1 expression, which induced mitochondrial fission and Cyt C release [120]. In addition, the overexpression of lncRNA CISAL could inhibit the transcription of the breast cancer type 1 susceptibility gene. This caused a decrease in miR-593 and subsequently upregulated the level of mitochondrial fission factor which results in Cyt C release and apoptosis during cisplatin therapy in TSCC [121]. Therefore, the mitochondrial fission-induced apoptosis of cancer cells may be related to the promotion of Cyt C release [104,122]. 

**Figure 4:**
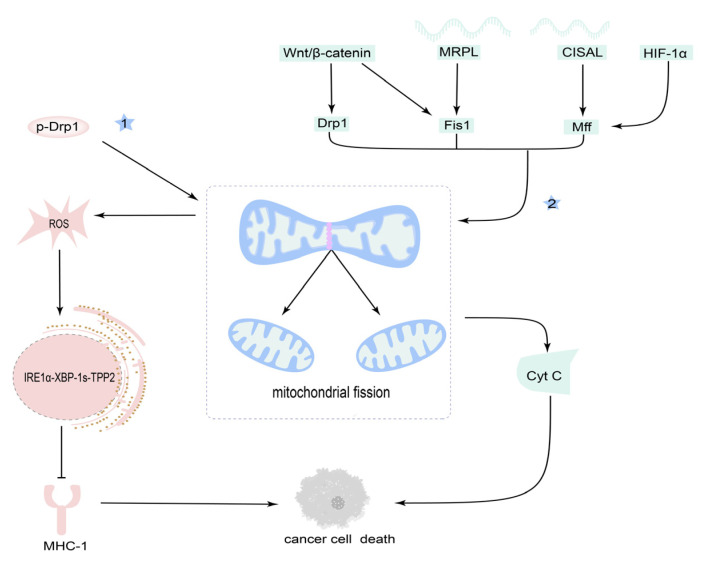
Targeting mitochondrial fission for OSCC therapy. (1) Inhibition of mitochondrial fission can cause cancer cell death by the upregulation of the major histocompatibility complex-I (MHC-I) in cancer cells. (2) Activation of mitochondrial fission can cause cancer cell death by the release of Cyt C from mitochondria.

These studies indicate that mitochondrial fission is a promising target in OSCC therapy (Figure 4), but the underlying mechanisms are not very clear, just as it not only serves as a promoter but plays as an objector in OSCC treatment.

## 4. Mitochondria in OSCC Therapy Resistance

Although mitochondria are beneficial to cancer treatment, abundant studies also found that they contributed to therapy resistance [17,123] in many cancers [124,125,126,127]. One reasonable explanation for this is that mitochondrial oncometabolites can upregulate the nuclear factor erythroid 2-related factor 2 pathway, therefore inhibiting the immunotherapy or promoting angiogenesis [36]. When it comes to OSCC, similar to therapeutic mechanisms, the therapeutic resistance mechanisms of mitochondria are also distinct. We will expound as follow. 

### 4.1. Mitophagy

Mitophagy, a process of selective mitochondrial autophagy, can eliminate dysfunctional mitochondria by forming autophagosomes for degradation, thereby protecting cells from death [46,128]. Obviously, mitophagy plays a significant role in resistance. For example, melatonin enhanced the effect of rapamycin in OSCC cells by inhibiting the AKT pathway and promoting the formation of mtROS. Nevertheless, when the concentration of melatonin was 1 mmol/L, the level of ROS did not increase, which was partly due to mitophagy [129]. Therefore, we should consider the impact of mitophagy when using therapies that destroy mitochondria via ROS generation. In addition to ROS, nutrient starvation was another condition that could promote mitophagy and protect OSCC from death via activating AMPK and inhibiting mTOR [130]. 

Obviously, restraining mitophagy is beneficial for OSCC treatment. For example, melatonin with verteporfin could reduce the expression of the key mitophagy regulators Parkin and PTEN-induced putative kinase 1 (PINK1) in the CSCs of OSCC, which induces the MAP in CSCs because damaged mitochondria caused by mtROS cannot be removed by mitophagy [131]. Indeed, mitophagy contributes to the survival of CSCs, which may be because mitophagy could remove damaged mitochondria and maintain moderate ROS levels to prevent programmed cell death [132]. Mitophagy could also maintain stemness by facilitating the expression of CSCs-related transcription factors including NANOG [133]. For example, mitophagy was higher in the cisplatin-resistant OSCC cells with higher CD44 and β-catenin expression, while the inhibition of autophagy could reduce the expression of CD44 and β-catenin [134]. However, this study did not expound on the specific mechanism of why autophagy/mitophagy could regulate the stemness of cisplatin-resistant OSCC cells. In addition, inhibiting lysosome function could also cause the death of drug-resistant OSCC cells, although the expression of the mitophagy-dependent proteins BNIP3 and PINK1 was increased because lysosomes were involved in the mitophagy process [135].

Interestingly, similar to autophagy, which is a double-edged sword in cancer, enhanced mitophagy is also beneficial to OSCC therapy. For example, zinc oxide nanoparticles (NPs) can induce mitochondrial damage and ROS in CAL-27 cells, which results in excessive PINK1/Parkin-mediated mitophagy, ultimately causing apoptosis [136]. 

**Figure 5:**
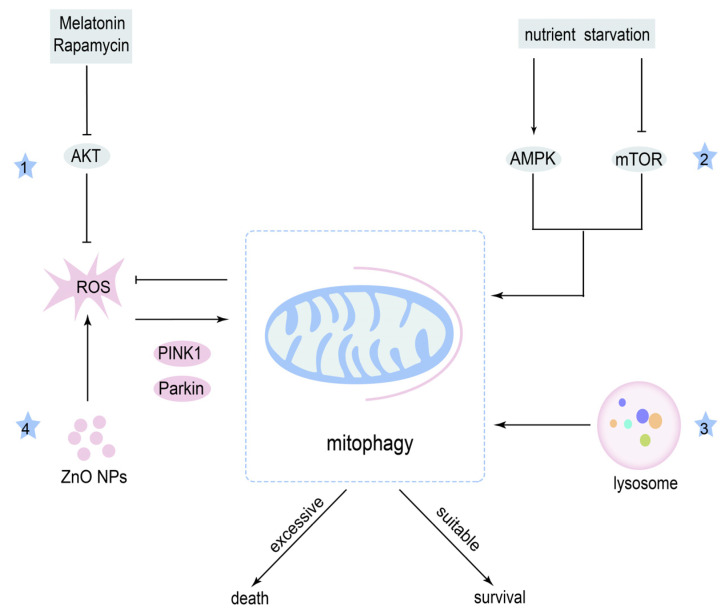
Mechanism of mitophagy in OSCC therapy and resistance. Mitophagy can reduce the influence of (1) ROS and (2) nutrient deficiency, thereby contributing to the survival of OSCC cells. Inhibiting the function of (3) lysosomes or excessive mitophagy induced by (4) Zinc oxide NPs (ZnO NPs) can cause OSCC cell death.

That evidence demonstrates that mitophagy has a double role: it protects cancer cells from death by removing dysfunctional mitochondria under unusual conditions, while excessive mitophagy causes tumor cell death by degrading a high number of mitochondria (Figure 5). Undoubtedly, relevant studies are limited, therefore, more studies of mitophagy in OSCC are urgently needed.

### 4.2. Mitochondrial MicroRNAs and Mitochondrial DNA

Mitochondrial microRNAs (mitomiRs), which originate in the nucleus or from mtDNA, are microRNAs located in the mitochondria that play a crucial role in the regulation of mitochondrial function [137]. 

Studies have found that mitomiRs can cause therapeutic resistance in OSCC. For example, the decreased expression of mitomiR-5787 can cause resistance to cisplatin in TSCC by shifting metabolism from OXPHOS to aerobic glycolysis; this in turn can induce greater lactate production, resulting in a low-pH environment [138]. In contrast, the increased expression of mitomiR-2392 could lead to cisplatin resistance in TSCC mediated by inhibiting mitochondrial OXPHOS gene transcription and promoting the expression of the glycolytic enzymes hexokinase 2 and PKM2; this results in OXPHOS downregulation and glycolysis upregulation, and thus, resistance [139]. In addition, microRNA-31 can drive OSCC invasion by destroying mitochondria and causing subsequent aerobic glycolysis [140]. Therefore, targeting microRNAs is another way to treat OSCC. For example, delivering microRNA-125 with ellagic acid to mitochondria could promote TSCC cell death by reducing antiapoptotic protein expression and increasing proapoptotic protein expression [141]. In addition, the overexpression of microRNA-378 could decrease the Bcl-2/Bax ratio by inactivating the phosphatidylinositol 3-kinase (PI3K)/AKT signaling pathway, which induces the MAP of OSCC [142]. 

In addition to mitomiRs, mtDNA is another type of nucleic acid that is present in mitochondria [143,144]. According to recent studies, the role of mtDNA in the therapeutic resistance in OSCC was mainly in two areas. First is the mtDNA copy number. For example, superabundant mtDNA contributed to the generation of healthy mitochondria and thus, supported the survival of HNSCC cells under radiation [145]. In contrast, low amounts of mtDNA may lead to a reduction in intracellular ROS production induced by cisplatin, thereby limiting its efficacy in OSCC cells [146]. The theoretical explanation for this paradoxical phenomenon may be that either an extremely high or a low mtDNA copy number was correlated with an increased risk of HNSCC [147]. Second, mtDNA mutations are common in cancers, including OSCC [148,149], and a prospective study found that mtDNA mutations were associated with worse outcomes in OSCC patients [150]. Similar to mitomiRs, mtDNA regulation can also produce antitumor effects [151]. For example, the connective tissue growth factor decreased the mtDNA copy number by the ubiquitin-mediated degradation of mitochondrial transcription Factor A which in turn caused mitochondrial biogenesis reduction, resulting in a decrease in ATP and the inhibition of OSCC cell migration and invasion [152].

**Figure 6:**
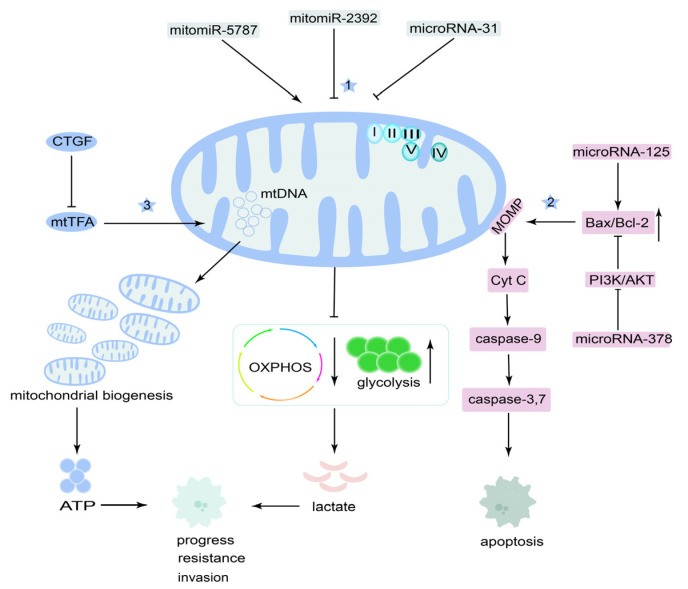
Mechanism of mitochondrial microRNA (mitomiRs) and mitochondrial DNA (mtDNA) in OSCC therapy and resistance. (1) Reducing oxidative phosphorylation (OXPHOS) and upregulating glycolysis via mitomiRs or microRNAs can induce lactate production, which causes resistance and invasion. (2) Regulating microRNAs can cause mitochondrial apoptosis pathways. (3) Upregulated mtDNA content can promote cancer progression.

From these studies, we know that both mitomiRs and mtDNA are related to the therapy and resistance of OSCC (Figure 6), but relative studies are limited; the reason may be because of our deficient knowledge of mitomiRs and mtDNA and technological limits.

### 4.3. Tumor Microenvironment

The role of mitochondria in therapeutic resistance is not only related to the tumor cells but also components of the TME, such as immune cells and fibroblasts [22].

The function of immune cells is closely related to mitochondria [153]. In addition, changes in mitochondria within tumor cells can also influence immune cell function. For instance, the overexpression of mitochondrial serine hydroxymethyltransferase 2 (SHMT2) was associated with advanced pathological grade and recurrence of OSCC [154]. Specifically, the expression of programmed cell death-ligand 1, V-type immunoglobulin domain-containing suppressor and B7-H4, in the TME, was positively correlated with SHMT2, which established an immunosuppressive status in OSCC [154]. Indeed, silencing SHMT2 inhibited OSCC progression [155]. In addition, mitochondrial Lon protein (Lon) could also inhibit OSCC immunotherapy. Mechanistically, Lon could promote ROS production to damage mtDNA, which caused the release of mtDNA into the cytosol and triggered interferon production via the cGAS-STING-TBK pathway. In this manner, the levels of programmed cell death-ligand 1 and indoleamine 2,3-dioxygenase 1 were upregulated, which inhibited T-cell activation. Moreover, Lon could induce the production of extracellular vesicles carrying programmed cell death-ligand 1 and mtDNA, which weakened both innate and T-cell immunity in the TME [156], and another group found that the mitochondrial Lon–ROS axis might promote OSCC progression and metastasis via inflammatory cytokine-induced M2 macrophage polarization [157].

In addition to immune cells, CAFs also interact with mitochondria in tumor cells [158]. First, CAFs could promote tumor progression in OSCC by providing lactate, which was a major source of the tricarboxylic acid cycle intermediates in many cancer cells [159]. Indeed, our previous study found that both primary CAFs and microvesicle-activated CAFs underwent metabolic reprogramming and produced more lactate, which was absorbed by OSCC cells to support their progression [160]. Similarly, the overexpression of integrin beta 2 could activate the PI3K/AKT/mTOR pathway to heighten the production of lactate in CAFs. This lactate was used to generate ATP in mitochondria of OSCC cells, which promoted OSCC proliferation [41]. Second, CAFs contribute to the stem-like properties of OSCC cells. For example, one of our studies found that lactate produced by CAFs could activate OXPHOS activity in OSCC and promote the organoid-forming ability of CSCs [161]. Finally, fibroblasts can prevent cancer cell death. For instance, when cocultured with OSCC cells, fibroblasts could rescue OSCC cells from growth inhibition and apoptosis induced by metformin because fibroblasts could maintain the MMP by inhibiting AMPK activity, which could upregulate the Bax/Bcl-2 ratio [21]. Interestingly, mitochondria could be transferred from fibroblasts to OSCC cells, resulting in a reduction in ROS levels in OSCC cells; thereby impairing the therapeutic effect of the drug that could upregulate the level of intracellular ROS [162]. Moreover, the mitochondrial transfer also occurs between cancer cells and immune cells [163].

**Figure 7:**
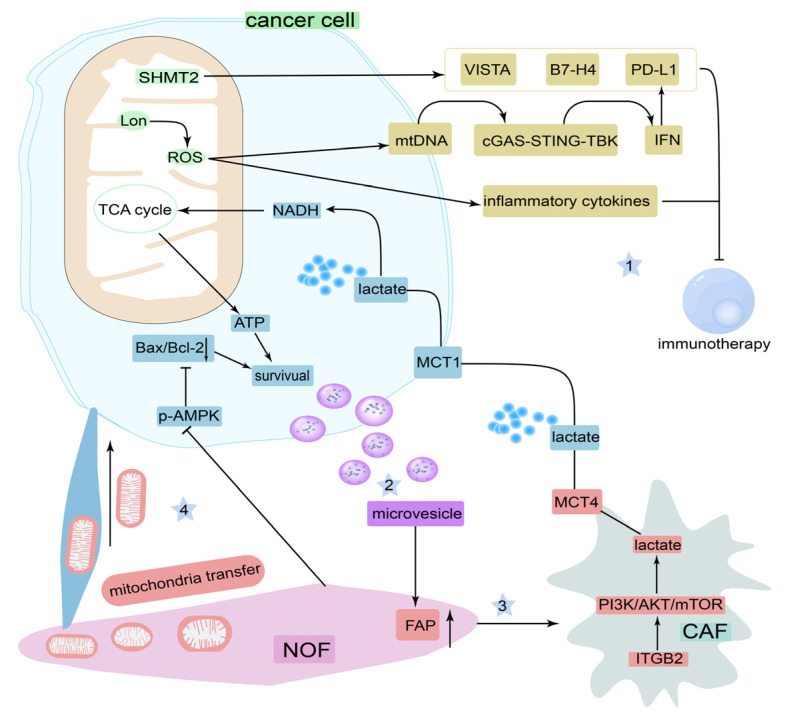
Communication between mitochondria of OSCC cells and immune cells or fibroblasts of the TME. (1) mitochondria suppress immunotherapy via the expression of serine hydroxymethyltransferase 2 (SHMT2) and mitochondrial Lon protein (Lon). (2) OSCC cells cause the transformation of normal fibroblasts (NOFs) to cancer-associated fibroblasts (CAFs) by microvesicles. (3) CAFs promote OSCC survival by providing lactate. (4) NOFs contribute to OSCC cell survival by inhibiting AMPK activity and by transferring mitochondria to OSCC cells.

Undoubtedly, the communication between mitochondria in tumor cells and the TME has a complex role in the therapeutic resistance observed in OSCC (Figure 7); however, relevant studies are limited, and more research should focus on this area.

## 5. Nanoparticles in Mitochondria-Targeted Therapy in OSCC

**Figure 8:**
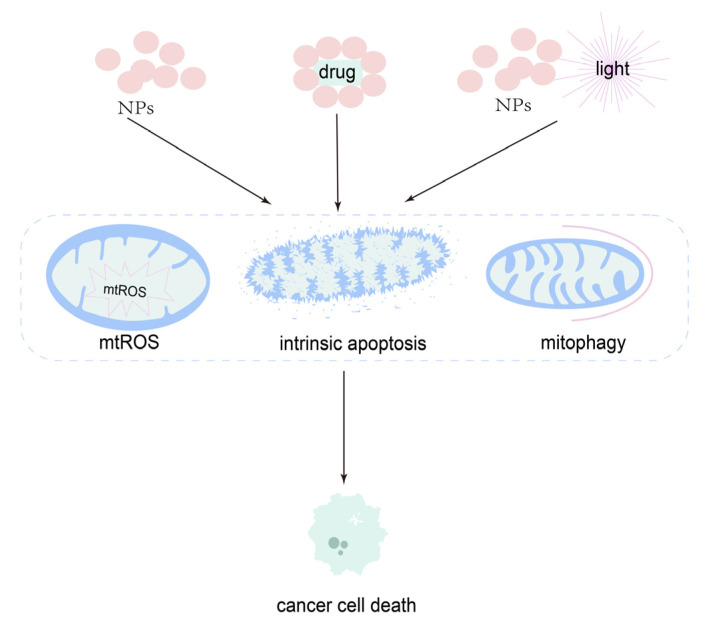
Mechanism of NPs targeting mitochondria as a therapy for OSCC. NPs alone, NPs loaded with drugs, and NPs plus light can induce cancer cell death via mtROS, the mitochondrial apoptosis pathway, and mitophagy.

Many therapeutic strategies have been applied in OSCC. However, the results are not satisfactory. Considering the limitations of surgery and radiotherapy, chemotherapy cannot be neglected [164]. Indeed, many synthetic chemicals have been applied to treat cancer by effecting mitochondrial function [58,165,166]. Of note, in the last decades, natural compounds have attracted particular interest in this field. For example, phenethyl isothiocyanates, which could lead to the mitochondrial cell death process, and gossypol, which could inhibit Bcl-2 and Bcl-xl, are undergoing clinical trials [167]. In addition, other natural compounds coming from Chinese herbal medicine, vegetable and animal, including resveratrol, curcumin and toxicarioside, can also influence mitochondrial function [168,169]. However, sometimes, low solubility, poor bioavailability and the side effects of harming normal tissues are the shortcomings of chemotherapy [164]. Fortunately, nanotechnology-based nanomedicine provides an encouraging solution [170,171]. Nanomedicine is the application of nanotechnology, which is related to materials belonging to the size range of 1–100 nm, in the medical field [172,173]. Nanotechnology plays an extensive and important role in the diagnosis [174], detection of metastasis and treatment of cancer [175]. For example, NPs could not only outline the primary and metastatic OSCC but could show a therapeutical effect [176], and could be used as a probe for the infrared fluorescence imaging of OSCC [177]. Therefore, nanomedicine, especially NPs, has extensive use in OSCC. Interestingly, NPs can also target mitochondria [23,178]. According to the articles we found, the application of NPs to target mitochondria in OSCC could be divided into three groups: NPs alone, NPs loaded with drugs and NPs with light stimulus-responsive cancer therapies (Figure 8).

First, inorganic NPs can destroy OSCC cells by impairing their mitochondria. For example, superparamagnetic iron oxide NPs could induce mtROS and Cyt C release, resulting in the apoptosis of OSCC cells [179]. A similar phenomenon was observed in TSCC cells [180]. In addition, zinc oxide NPs could induce MAP by inducing superoxide production in gingival squamous cell carcinoma cells in vitro and in vivo, while normal cells were unaffected [181]. Furthermore, zinc oxide NPs could cause excess PINK1/Parkin-mediated mitophagy by upregulating mtROS, which resulted in OSCC cell death [136], and quinacrine-based gold hybrid nanoparticles not only showed promotion of MAP by increasing Bax and ROS, and reducing Bcl-2, but could impair inflammation reaction by reducing proinflammatory cytokines, such as interleukin-6 and interleukin-8, and could suppress angiogenesis via downregulating angiopoietin-1, vascular endothelial growth factor and matrix metalloproteinase-2 in the CSCs of OSCC [182]. 

Moreover, NPs can also exert their anticancer effects as drug carriers [183]. For example, the γ-polyglutamic acid-gefitinib/curcumin NPs could induce mitochondrial Cyt C release and caspase-3 activation in OSCC cells. Furthermore, this nanocomposite observably restrained the tumor size in OSCC mice compared with the free gefitinib/curcumin-treated group, which may be because NPs can be more easily absorbed into tumor cells, thereby improving the concentration of gefitinib and curcumin in OSCC cells [184]. Another study found that phloretin-loaded chitosan NPs could induce the MAP in oral cancer and that the IC50 was lower than that of phloretin alone. The difference might be caused by the electrostatic ionic interaction between phloretin-loaded chitosan NPs and tumor cells [185]. 

Light stimulus-responsive cancer therapies, including photodynamic therapy, and photothermal therapy, are attractive methods for OSCC treatment [186]. NPs, interestingly, have a synergistic effect with these therapies when targeting mitochondria. For instance, chitosan-5-aminolevulinic acid-glioblastoma-amplified sequence gene plasmid DNA NPs with photodynamic therapy could better inhibit the growth of OSCC cells by inducing mtROS production compared with 5-aminolevulinic acid–photodynamic therapy treatment [187]. Meaningfully, after the quinacrine–gold hybrid nanoparticle was absorbed by the CSCs of OSCC, near-infrared radiation could activate it, which resulted in heat stress and subsequently increased Bax and Cyt c and decreased Bcl-2, causing the MAP [188]. 

Obviously, NPs in mitochondria-targeted therapy for OSCC show a promising future, however, different challenges need to be solved. For instance, nanotechnology-based materials need to overcome physiological barriers, such as the TME, to target cancer cells [189], and the heterogeneity of tumors also needs to be considered [190].

## 6. Conclusions and Future Directions

Mitochondria are a double-edged sword in OSCC therapy. On a positive note, targeting mitochondria for therapy can be achieved by (1) regulating the Bcl-2 family to initiate the MAP, (2) improving the level of ROS to induce mitochondria-related cell death, and (3) regulating mitochondrial fission to promote cell death. On a negative note, mitochondria cause therapeutic resistance through (1) mitophagy, which can clear damaged mitochondria; (2) mitomiR and mtDNA, which can cause metabolic reprogramming; and (3) the TME, which interacts with mitochondria in OSCC cells. Indeed, NPs targeting mitochondria by the above mechanisms is a promising approach to treating OSCC. 

However, many questions still warrant further exploration between OSCC and mitochondria under this paper: (1) Can the regulation of organelles other than the ER be used to induce the MAP? (2) Can lncRNAs regulate mitomiRs and mtDNA? (3) How do mitomiRs and mtDNA interact? (4) What is the interaction between cells other than immune cells and fibroblasts in the TME and mitochondria of tumor cells? In addition, some questions, beyond this review, also need to be answered. First, abundant studies have found OSCC with abnormal metabolism [191,192], however, the role of mitochondria in those processes is indistinct. Second, mitochondria can regulate the epigenetics of nuclear DNA [193], but how the nucleus regulates the epigenetics of mtDNA is indeterminate [194]. Third, senescent cells have been regarded as a hallmark of cancer [193], while the role of mitochondria in cell aging has been proved [195], and four, mitochondria have a tight relationship with inflammation [196], while inflammation significantly promotes all stages of tumorigenesis [197]. Therefore, investigating the link between mitochondria with OSCC-associated senescent cells or the OSCC-associated process of inflammation is interesting and meaningful.

Finally, mitochondrial fission-relative proteins, such as Drp1 and fission protein 1, are overexpressed in salivary adenoid cystic carcinoma [198], and MOMP might play a significant role in inducing salivary adenoid cystic carcinoma cell apoptosis [199]. These studies imply that mitochondria may also play a similar role in those pathologic types that, like adenocarcinoma and sarcoma, exist in the oral cavity. Obviously, relative studies are very limited and more studies about the mitochondrial role in those cancer types are worth performing, even though those cancer types account for only a small proportion of oral cancers.

## References

[1] Meng X., Lou Q., Yang W., Wang Y., Chen R., Wang L., Xu T., Zhang L. (2021). The role of non-coding RNAs in drug resistance of oral squamous cell carcinoma and therapeutic potential. Cancer Commun..

[2] Chai A.W.Y., Lim K.P., Cheong S.C. (2020). Translational genomics and recent advances in oral squamous cell carcinoma. Semin. Cancer Biol..

[3] Thomson P.J. (2018). Perspectives on oral squamous cell carcinoma prevention—Proliferation, position, progression and prediction. J. Oral Pathol. Med..

[4] Ng J.H., Iyer N.G., Tan M.-H., Edgren G. (2016). Changing epidemiology of oral squamous cell carcinoma of the tongue: A global study. Head Neck.

[5] Johnson D.E., Burtness B., Leemans C.R., Lui V.W.Y., Bauman J.E., Grandis J.R. (2020). Head and neck squamous cell carcinoma. Nat. Rev. Dis. Primers.

[6] Zanoni D.K., Montero P.H., Migliacci J.C., Shah J.P., Wong R.J., Ganly I., Patel S.G. (2019). Survival outcomes after treatment of cancer of the oral cavity (1985–2015). Oral Oncol..

[7] Zeng W., Zhang P. (2021). Resistance and recurrence of malignancies after CAR-T cell therapy. Exp. Cell Res..

[8] Mahato R., Tai W., Cheng K. (2011). Prodrugs for improving tumor targetability and efficiency. Adv. Drug Deliv. Rev..

[9] Kummer E., Ban N. (2021). Mechanisms and regulation of protein synthesis in mitochondria. Nat. Rev. Mol. Cell Biol..

[10] Barba-Aliaga M., Alepuz P. (2022). Role of eIF5A in Mitochondrial Function. Int. J. Mol. Sci..

[11] Porporato P.E., Filigheddu N., Pedro J.M.B.-S., Kroemer G., Galluzzi L. (2018). Mitochondrial metabolism and cancer. Cell Res..

[12] Chou G.-L., Peng S.-F., Liao C., Ho H.-C., Lu K.-W., Lien J.-C., Fan M.-J., La K.-C., Chung J.-G. (2018). Casticin impairs cell growth and induces cell apoptosis via cell cycle arrest in human oral cancer SCC-4 cells. Environ. Toxicol..

[13] Chen H., Li L., Lu Y., Shen Y., Zhang M., Ge L., Wang M., Yang J., Tian Z., Tang X. (2020). Azoxystrobin Reduces Oral Carcinogenesis by Suppressing Mitochondrial Complex III Activity and Inducing Apoptosis. Cancer Manag. Res..

[14] Lei X., Lin H., Wang J., Ou Z., Ruan Y., Sadagopan A., Chen W., Xie S., Chen B., Li Q. (2022). Mitochondrial fission induces immunoescape in solid tumors through decreasing MHC-I surface expression. Nat. Commun..

[15] Kraus F., Roy K., Pucadyil T.J., Ryan M.T. (2021). Function and regulation of the divisome for mitochondrial fission. Nature.

[16] Genovese I., Carinci M., Modesti L., Aguiari G., Pinton P., Giorgi C. (2021). Mitochondria: Insights into Crucial Features to Overcome Cancer Chemoresistance. Int. J. Mol. Sci..

[17] Guerra F., Arbini A.A., Moro L. (2017). Mitochondria and cancer chemoresistance. Biochim. Biophys. Acta (BBA)—Bioenerg..

[18] Guerra-Librero A., Fernandez-Gil B., Florido J., Martinez-Ruiz L., Rodríguez-Santana C., Shen Y.-Q., García-Verdugo J., López-Rodríguez A., Rusanova I., Quiñones-Hinojosa A. (2021). Melatonin Targets Metabolism in Head and Neck Cancer Cells by Regulating Mitochondrial Structure and Function. Antioxidants.

[19] Purohit P.K., Saini N. (2020). Mitochondrial microRNA (MitomiRs) in cancer and complex mitochondrial diseases: Current status and future perspectives. Cell. Mol. Life Sci..

[20] Hsieh Y.-T., Tu H.-F., Yang M.-H., Chen Y.-F., Lan X.-Y., Huang C.-L., Chen H.-M., Li W.-C. (2021). Mitochondrial genome and its regulator TFAM modulates head and neck tumourigenesis through intracellular metabolic reprogramming and activation of oncogenic effectors. Cell Death Dis..

[21] Zhang Z., Liang K.X., Fan Y., Gao Z., Bindoff L.A., Costea D.E., Li L. (2019). Fibroblasts rescue oral squamous cancer cell from metformin-induced apoptosis via alleviating metabolic disbalance and inhibiting AMPK pathway. Cell Cycle.

[22] Han Y., Cho U., Kim S., Park I.S., Cho J.H., Dhanasekaran D.N., Song Y.S. (2018). Tumour microenvironment on mitochondrial dynamics and chemoresistance in cancer. Free Radic. Res..

[23] Mani S., Swargiary G., Tyagi S., Singh M., Jha N.K., Singh K.K. (2021). Nanotherapeutic approaches to target mitochondria in cancer. Life Sci..

[24] Zong W.-X., Rabinowitz J.D., White E. (2016). Mitochondria and Cancer. Mol. Cell.

[25] Wu Z., Sainz A.G., Shadel G.S. (2021). Mitochondrial DNA: Cellular genotoxic stress sentinel. Trends Biochem. Sci..

[26] Varela-López A., Vera-Ramírez L., Giampieri F., Navarro-Hortal M.D., Forbes-Hernández T.Y., Battino M., Quiles J.L. (2021). The central role of mitochondria in the relationship between dietary lipids and cancer progression. Semin. Cancer Biol..

[27] Wallace D.C. (2012). Mitochondria and cancer. Nat. Rev. Cancer.

[28] Ghosh P., Vidal C., Dey S., Zhang L. (2020). Mitochondria Targeting as an Effective Strategy for Cancer Therapy. Int. J. Mol. Sci..

[29] Xu X.D., Shao S.X., Jiang H.P., Cao Y.W., Wang Y.H., Yang X.C., Wang Y.L., Wang X.S., Niu H.T. (2015). Warburg Effect or Reverse Warburg Effect? A Review of Cancer Metabolism. Oncol. Res. Treat..

[30] Vaupel P., Multhoff G. (2020). Revisiting the Warburg effect: Historical dogma versus current understanding. J. Physiol..

[31] Hanahan D. (2022). Hallmarks of Cancer: New Dimensions. Cancer Discov..

[32] Missiroli S., Perrone M., Genovese I., Pinton P., Giorgi C. (2020). Cancer metabolism and mitochondria: Finding novel mechanisms to fight tumours. eBioMedicine.

[33] Badrinath N., Yoo S.Y. (2018). Mitochondria in cancer: In the aspects of tumorigenesis and targeted therapy. Carcinogenesis.

[34] Sessions D.T., Kashatus D.F. (2021). Mitochondrial dynamics in cancer stem cells. Cell. Mol. Life Sci..

[35] Scheid A.D., Beadnell T.C., Welch D.R. (2020). Roles of mitochondria in the hallmarks of metastasis. Br. J. Cancer.

[36] van der Merwe M., van Niekerk G., Fourie C., du Plessis M., Engelbrecht A.-M. (2021). The impact of mitochondria on cancer treatment resistance. Cell Oncol..

[37] Hou X.-S., Wang H.-S., Mugaka B.P., Yang G.-J., Ding Y. (2018). Mitochondria: Promising organelle targets for cancer diagnosis and treatment. Biomater. Sci..

[38] Hu Q., Peng J., Chen X., Li H., Song M., Cheng B., Wu T. (2018). Obesity and genes related to lipid metabolism predict poor survival in oral squamous cell carcinoma. Oral Oncol..

[39] de Mattos S., Diel L., Bittencourt L., Schnorr C., Gonçalves F., Bernardi L., Lamers M. (2021). Glycolytic pathway candidate markers in the prognosis of oral squamous cell carcinoma: A systematic review with meta-analysis. Braz. J. Med. Biol. Res..

[40] Wu R., Zuo W., Xu X., Bi L., Zhang C., Chen H., Liu H. (2021). MCU That Is Transcriptionally Regulated by Nrf2 Augments Malignant Biological Behaviors in Oral Squamous Cell Carcinoma Cells. BioMed. Res. Int..

[41] Zhang X., Dong Y., Zhao M., Ding L., Yang X., Jing Y., Song Y., Chen S., Hu Q., Ni Y. (2020). ITGB2-mediated metabolic switch in CAFs promotes OSCC proliferation by oxidation of NADH in mitochondrial oxidative phosphorylation system. Theranostics.

[42] Gao J., Zhao Y., Li T., Gan X., Yu H. (2022). The Role of PKM2 in the Regulation of Mitochondrial Function: Focus on Mitochondrial Metabolism, Oxidative Stress, Dynamic, and Apoptosis. PKM2 in Mitochondrial Function. Oxidative Med. Cell Longev..

[43] Kurihara-Shimomura M., Sasahira T., Nakashima C., Kuniyasu H., Shimomura H., Kirita T. (2018). The Multifarious Functions of Pyruvate Kinase M2 in Oral Cancer Cells. Int. J. Mol. Sci..

[44] Ashton T.M., McKenna W.G., Kunz-Schughart L.A., Higgins G.S. (2018). Oxidative Phosphorylation as an Emerging Target in Cancer Therapy. Clin. Cancer Res..

[45] Vasan K., Werner M., Chandel N.S. (2020). Mitochondrial Metabolism as a Target for Cancer Therapy. Cell Metab..

[46] Panigrahi D.P., Praharaj P.P., Bhol C.S., Mahapatra K.K., Patra S., Behera B.P., Mishra S.R., Bhutia S.K. (2019). The emerging, multifaceted role of mitophagy in cancer and cancer therapeutics. Semin. Cancer Biol..

[47] Burke P.J. (2017). Mitochondria, Bioenergetics and Apoptosis in Cancer. Trends Cancer.

[48] Bock F.J., Tait S.W.G. (2020). Mitochondria as multifaceted regulators of cell death. Nat. Rev. Mol. Cell Biol..

[49] Gan B. (2021). Mitochondrial regulation of ferroptosis. J. Cell Biol..

[50] Tang D., Chen X., Kroemer G. (2022). Cuproptosis: A copper-triggered modality of mitochondrial cell death. Cell Res..

[51] Faizan I., Ahmad T. (2021). Altered mitochondrial calcium handling and cell death by necroptosis: An emerging paradigm. Mitochondrion.

[52] Bernard N.J. (2021). Mitochondria control pyroptosis. Nat. Immunol..

[53] Abate M., Festa A., Falco M., Lombardi A., Luce A., Grimaldi A., Zappavigna S., Sperlongano P., Irace C., Caraglia M. (2020). Mitochondria as playmakers of apoptosis, autophagy and senescence. Semin. Cell Dev. Biol..

[54] Carneiro B.A., El-Deiry W.S. (2020). Targeting apoptosis in cancer therapy. Nat. Rev. Clin. Oncol..

[55] Diepstraten S.T., Anderson M.A., Czabotar P.E., Lessene G., Strasser A., Kelly G.L. (2021). The manipulation of apoptosis for cancer therapy using BH3-mimetic drugs. Nat. Rev. Cancer.

[56] Messmer M.N., Snyder A.G., Oberst A. (2019). Comparing the effects of different cell death programs in tumor progression and immunotherapy. Cell Death Differ..

[57] Fontana F., Limonta P. (2021). The multifaceted roles of mitochondria at the crossroads of cell life and death in cancer. Free Radic. Biol. Med..

[58] Adams J., Cory S. (2018). The BCL-2 arbiters of apoptosis and their growing role as cancer targets. Cell Death Differ..

[59] Cowan A.D., Smith N.A., Sandow J.J., Kapp E.A., Rustam Y.H., Murphy J.M., Brouwer J.M., Bernardini J.P., Roy M.J., Wardak A.Z. (2020). BAK core dimers bind lipids and can be bridged by them. Nat. Struct. Mol. Biol..

[60] Cosentino K., García-Sáez A.J. (2016). Bax and Bak Pores: Are We Closing the Circle?. Trends Cell Biol..

[61] Ow Y.-L.P., Green D.R., Hao Z., Mak T.W. (2008). Cytochrome c: Functions beyond respiration. Nat. Rev. Mol. Cell Biol..

[62] Kalkavan H., Green D. (2017). MOMP, cell suicide as a BCL-2 family business. Cell Death Differ..

[63] Hammouda M.B., Ford A.E., Liu Y., Zhang J.Y. (2020). The JNK Signaling Pathway in Inflammatory Skin Disorders and Cancer. Cells.

[64] Hsieh M.-Y., Lo Y.-S., Lin C.-C., Chuang Y.-C., Chen M.-K., Chou M.-C. (2020). Modulating effect of Coronarin D in 5-fluorouracil resistance human oral cancer cell lines induced apoptosis and cell cycle arrest through JNK1/2 signaling pathway. Biomed. Pharmacother..

[65] Huang C.-F., Liu S.-H., Ho T.-J., Lee K.-I., Fang K.-M., Lo W.-C., Liu J.-M., Wu C.-C., Su C.-C. (2022). Quercetin induces tongue squamous cell carcinoma cell apoptosis via the JNK activation-regulated ERK/GSK-3α/β-mediated mitochondria-dependent apoptotic signaling pathway. Oncol. Lett..

[66] Liu Y.-T., Hsieh M.-J., Lin J.-T., Chen G., Lin C.-C., Lo Y.-S., Chuang Y.-C., Hsi Y.-T., Chen M.-K., Chou M.-C. (2019). Coronarin D induces human oral cancer cell apoptosis though upregulate JNK1/2 signaling pathway. Environ. Toxicol..

[67] Chen C., Yang J., Chen W., Lu C., Chiang J., Chiu H., Tsai S., Juan Y., Huang H., Way T. (2018). Ursolic acid elicits intrinsic apoptotic machinery by downregulating the phosphorylation of AKT/BAD signaling in human cisplatin-resistant oral cancer CAR cells. Oncol. Rep..

[68] Zhang Z., Lin R., Liu Z., Yan T., Xia Y., Zhao L., Lin F., Zhang X., Li C., Wang Y. (2019). Curcumin analog, WZ37, promotes G2/M arrest and apoptosis of HNSCC cells through Akt/mTOR inhibition. Toxicol. Vitr..

[69] Seo J.-H., Choi H.W., Oh H.-N., Lee M.-H., Kim E., Yoon G., Cho S.-S., Park S.-M., Cho Y.S., Chae J. (2018). Licochalcone D directly targets JAK2 to induced apoptosis in human oral squamous cell carcinoma. J. Cell Physiol..

[70] Zacks D.N., Zheng Q.-D., Bakhru R., Han Y., Miller J.W. (2004). FAS-Mediated Apoptosis and Its Relation to Intrinsic Pathway Activation in an Experimental Model of Retinal Detachment. Investig. Opthalmology Vis. Sci..

[71] Taylor R.C., Cullen S.P., Martin S.J. (2008). Apoptosis: Controlled demolition at the cellular level. Nat. Rev. Mol. Cell Biol..

[72] Cai J., Yi M., Tan Y., Li X., Li G., Zeng Z., Xiong W., Xiang B. (2021). Natural product triptolide induces GSDME-mediated pyroptosis in head and neck cancer through suppressing mitochondrial hexokinase-ΙΙ. J. Exp. Clin. Cancer Res..

[73] Dev A., Sardoiwala M.N., Kushwaha A.C., Karmakar S., Choudhury S.R. (2020). Genistein nanoformulation promotes selective apoptosis in oral squamous cell carcinoma through repression of 3PK-EZH2 signalling pathway. Phytomedicine.

[74] Yang H., Villani R.M., Wang H., Simpson M.J., Roberts M.S., Tang M., Liang X. (2018). The role of cellular reactive oxygen species in cancer chemotherapy. J. Exp. Clin. Cancer Res..

[75] Yin M., O’Neill L.A.J. (2021). The role of the electron transport chain in immunity. FASEB J..

[76] Sabharwal S.S., Schumacker P.T. (2014). Mitochondrial ROS in cancer: Initiators, amplifiers or an Achilles’ heel?. Nat. Rev. Cancer.

[77] Idelchik M.D.P.S., Begley U., Begley T.J., Melendez J.A. (2017). Mitochondrial ROS control of cancer. Semin. Cancer Biol..

[78] Rosini E., Pollegioni L. (2021). Reactive oxygen species as a double-edged sword: The role of oxidative enzymes in antitumor therapy. BioFactors.

[79] Nathan C., Cunningham-Bussel A. (2013). Beyond oxidative stress: An immunologist’s guide to reactive oxygen species. Nat. Rev. Immunol..

[80] Görlach A., Bertram K., Hudecova S., Krizanova O. (2015). Calcium and ROS: A mutual interplay. Redox Biol..

[81] Halestrap A.P., McStay G.P., Clarke S.J. (2002). The permeability transition pore complex: Another view. Biochimie.

[82] Simon H.-U., Haj-Yehia A., Levi-Schaffer F. (2000). Role of reactive oxygen species (ROS) in apoptosis induction. Apoptosis.

[83] Bauer T.M., Murphy E. (2020). Role of Mitochondrial Calcium and the Permeability Transition Pore in Regulating Cell Death. Circ. Res..

[84] Yu T., Hsieh C., Tang J., Lin L., Huang H., Wang H., Yeh Y., Chuang Y., Ou-Yang F., Chang H. (2020). Antimycin A shows selective antiproliferation to oral cancer cells by oxidative stress-mediated apoptosis andDNAdamage. Environ. Toxicol..

[85] Li D., Ueta E., Kimura T., Yamamoto T., Osaki T. (2004). Reactive oxygen species (ROS) control the expression of Bcl-2 family proteins by regulating their phosphorylation and ubiquitination. Cancer Sci..

[86] Orrenius S., Gogvadze V., Zhivotovsky B. (2015). Calcium and mitochondria in the regulation of cell death. Biochem. Biophys. Res. Commun..

[87] Li M.-H., Liao X., Li C., Wang T.-T., Sun Y.-S., Yang K., Jiang P.-W., Shi S.-T., Zhang W.-X., Zhang K. (2021). Lycorine hydrochloride induces reactive oxygen species-mediated apoptosis via the mitochondrial apoptotic pathway and the JNK signaling pathway in the oral squamous cell carcinoma HSC-3 cell line. Oncol. Lett..

[88] Chong S.J.F., Low I.C.C., Pervaiz S. (2014). Mitochondrial ROS and involvement of Bcl-2 as a mitochondrial ROS regulator. Mitochondrion.

[89] Han D., Antunes F., Canali R., Rettori D., Cadenas E. (2003). Voltage-dependent Anion Channels Control the Release of the Superoxide Anion from Mitochondria to Cytosol. J. Biol. Chem..

[90] Vo T.T.T., Liu J.-F., Wu C.-Z., Lin W.-N., Chen Y.-L., Lee I.-T. (2020). Surfactin from *Bacillus subtilis* induces apoptosis in human oral squamous cell carcinoma through ROS-regulated mitochondrial pathway. J. Cancer.

[91] Zhang Q., Cheng G., Pan J., Zielonka J., Xiong D., Myers C.R., Feng L., Shin S.S., Kim Y.H., Bui D. (2020). Magnolia extract is effective for the chemoprevention of oral cancer through its ability to inhibit mitochondrial respiration at complex I. Cell Commun. Signal..

[92] Redza-Dutordoir M., Averill-Bates D.A. (2016). Activation of apoptosis signalling pathways by reactive oxygen species. Biochim. Biophys. Acta (BBA)—Mol. Cell Res..

[93] Tang J.-Y., Wu K.-H., Wang Y.-Y., Farooqi A., Huang H.-W., Yuan S.F., Jian R.-I., Tsao L.-Y., Chen P.-A., Chang F.-R. (2020). Methanol Extract of *Usnea barbata* Induces Cell Killing, Apoptosis, and DNA Damage against Oral Cancer Cells through Oxidative Stress. Antioxidants.

[94] Wang H.-R., Tang J.-Y., Wang Y.-Y., Farooqi A.A., Yen C.-Y., Yuan S.-S.F., Huang H.-W., Chang H.-W. (2019). Manoalide Preferentially Provides Antiproliferation of Oral Cancer Cells by Oxidative Stress-Mediated Apoptosis and DNA Damage. Cancers.

[95] Saluja T.S., Kumar V., Agrawal M., Tripathi A., Meher R.K., Srivastava K., Gupta A., Singh A., Chaturvedi A., Singh S.K. (2020). Mitochondrial Stress–Mediated Targeting of Quiescent Cancer Stem Cells in Oral Squamous Cell Carcinoma. Cancer Manag. Res..

[96] Zorov D.B., Juhaszova M., Sollott S.J. (2006). Mitochondrial ROS-induced ROS release: An update and review. Biochim. Biophys. Acta (BBA)—Bioenerg..

[97] He W., Lai R., Lin Q., Huang Y., Wang L. (2019). Arglabin is a plant sesquiterpene lactone that exerts potent anticancer effects on human oral squamous cancer cells via mitochondrial apoptosis and downregulation of the mTOR/PI3K/Akt signaling pathway to inhibit tumor growth in vivo. J. Buon Off. J. Balk. Union Oncol..

[98] Martucciello S., Masullo M., Cerulli A., Piacente S. (2020). Natural Products Targeting ER Stress, and the Functional Link to Mitochondria. Int. J. Mol. Sci..

[99] Rossi A., Pizzo P., Filadi R. (2019). Calcium, mitochondria and cell metabolism: A functional triangle in bioenergetics. Biochim. Biophys. Acta Mol. Cell Res..

[100] Doghman M., Lalli E. (2019). ER-mitochondria interactions: Both strength and weakness within cancer cells. Biochim. Biophys. Acta.

[101] Madreiter-Sokolowski C.T., Thomas C., Ristow M. (2020). Interrelation between ROS and Ca2+ in aging and age-related diseases. Redox Biol..

[102] Moniruzzaman R., Rehman M.U., Zhao Q.-L., Jawaid P., Mitsuhashi Y., Sakurai K., Heshiki W., Ogawa R., Tomihara K., Saitoh J.-I. (2019). Combination of 5-aminosalicylic acid and hyperthermia synergistically enhances apoptotic cell death in HSC-3 cells due to intracellular nitric oxide/peroxynitrite generation. Cancer Lett..

[103] Ansari S.S., Sharma A.K., Soni H., Ali D.M., Tews B., König R., Eibl H., Berger M.R. (2018). Induction of ER and mitochondrial stress by the alkylphosphocholine erufosine in oral squamous cell carcinoma cells. Cell Death Dis..

[104] Trotta A.P., Chipuk J.E. (2017). Mitochondrial dynamics as regulators of cancer biology. Cell. Mol. Life Sci..

[105] Tilokani L., Nagashima S., Paupe V., Prudent J. (2018). Mitochondrial dynamics: Overview of molecular mechanisms. Essays Biochem..

[106] Liu R., Chan D.C. (2015). The mitochondrial fission receptor Mff selectively recruits oligomerized Drp1. Mol. Biol. Cell.

[107] Yu R., Jin S., Lendahl U., Nistér M., Zhao J. (2019). Human Fis1 regulates mitochondrial dynamics through inhibition of the fusion machinery. EMBO J..

[108] Kamerkar S.C., Kraus F., Sharpe A.J., Pucadyil T.J., Ryan M.T. (2018). Dynamin-related protein 1 has membrane constricting and severing abilities sufficient for mitochondrial and peroxisomal fission. Nat. Commun..

[109] Chiu Y.-H., Lin S.-C.A., Kuo C.-H., Li C.-J. (2021). Molecular Machinery and Pathophysiology of Mitochondrial Dynamics. Front. Cell Dev. Biol..

[110] Huang C.-Y., Chiang S.-F., Chen W.T.-L., Ke T.-W., Chen T.-W., You Y.-S., Lin C.-Y., Chao K.S.C. (2018). HMGB1 promotes ERK-mediated mitochondrial Drp1 phosphorylation for chemoresistance through RAGE in colorectal cancer. Cell Death Dis..

[111] Huang L., Luan T., Chen Y., Bao X., Huang Y., Fu S., Wang H., Wang J. (2018). LASS2 regulates invasion and chemoresistance via ERK/Drp1 modulated mitochondrial dynamics in bladder cancer cells. J. Cancer.

[112] Han Y., Kim B., Cho U., Park I.S., Kim S.I., Dhanasekaran D.N., Tsang B.K., Song Y.S. (2019). Mitochondrial fission causes cisplatin resistance under hypoxic conditions via ROS in ovarian cancer cells. Oncogene.

[113] Chang Y.-J., Chen K.-W., Chen L. (2020). Mitochondrial ROS1 Increases Mitochondrial Fission and Respiration in Oral Squamous Cancer Carcinoma. Cancers.

[114] Ghosh A., Chatterjee K., Chowdhury A.R., Barui A. (2020). Clinico-pathological significance of Drp1 dysregulation and its correlation to apoptosis in oral cancer patients. Mitochondrion.

[115] Li S., Wu Y., Ding Y., Yu M., Ai Z. (2018). CerS6 regulates cisplatin resistance in oral squamous cell carcinoma by altering mitochondrial fission and autophagy. J. Cell Physiol..

[116] Tsao Y.-C., Chang Y.-J., Wang C.-H., Chen L. (2020). Discovery of Isoplumbagin as a Novel NQO1 Substrate and Anti-Cancer Quinone. Int. J. Mol. Sci..

[117] Pearson H.E., Iida M., Orbuch R.A., McDaniel N.K., Nickel K.P., Kimple R.J., Arbiser J.L., Wheeler D.L. (2018). Overcoming Resistance to Cetuximab with Honokiol, A Small-Molecule Polyphenol. Mol. Cancer Ther..

[118] Wu K., Mao Y.-Y., Chen Q., Zhang B., Zhang S., Wu H.-J., Li Y. (2021). Hypoxia-induced ROS promotes mitochondrial fission and cisplatin chemosensitivity via HIF-1α/Mff regulation in head and neck squamous cell carcinoma. Cell Oncol..

[119] Ma C., Fan L., Wang J., Hao L., He J. (2019). Hippo/Mst1 overexpression induces mitochondrial death in head and neck squamous cell carcinoma via activating β-catenin/Drp1 pathway. Cell Stress Chaperon-.

[120] Tian T., Lv X., Pan G., Lu Y., Chen W., He W., Lei X., Zhang H., Liu M., Sun S. (2019). Long Noncoding RNA MPRL Promotes Mitochondrial Fission and Cisplatin Chemosensitivity via Disruption of Pre-miRNA Processing. Clin. Cancer Res..

[121] Fan S., Tian T., Lv X., Lei X., Yang Z., Liu M., Liang F., Li S., Lin X., Lin Z. (2020). lncRNA CISAL Inhibits BRCA1 Transcription by Forming a Tertiary Structure at Its Promoter. iScience.

[122] Westermann B. (2010). Mitochondrial fusion and fission in cell life and death. Nat. Rev. Mol. Cell Biol..

[123] Lyakhovich A., Lleonart M.E. (2015). Bypassing Mechanisms of Mitochondria-Mediated Cancer Stem Cells Resistance to Chemo- and Radiotherapy. Oxidative Med. Cell Longev..

[124] Fernandez H.R., Gadre S.M., Tan M., Graham G.T., Mosaoa R., Ongkeko M.S., Kim K.A., Riggins R.B., Parasido E., Petrini I. (2018). The mitochondrial citrate carrier, SLC25A1, drives stemness and therapy resistance in non-small cell lung cancer. Cell Death Differ..

[125] Li Y., Li Z. (2021). Potential Mechanism Underlying the Role of Mitochondria in Breast Cancer Drug Resistance and Its Related Treatment Prospects. Front. Oncol..

[126] Shen L., Xia M., Zhang Y., Luo H., Dong D., Sun L. (2021). Mitochondrial integration and ovarian cancer chemotherapy resistance. Exp. Cell Res..

[127] Fu Y., Ricciardiello F., Yang G., Qiu J., Huang H., Xiao J., Cao Z., Zhao F., Liu Y., Luo W. (2021). The Role of Mitochondria in the Chemoresistance of Pancreatic Cancer Cells. Cells.

[128] Onishi M., Yamano K., Sato M., Matsuda N., Okamoto K. (2021). Molecular mechanisms and physiological functions of mitophagy. EMBO J..

[129] Shen Y.-Q., Guerra-Librero A., Fernandez-Gil B.I., Florido J., García-López S., Ruiz L.M., Mendivil-Perez M., Soto-Mercado V., Acuña-Castroviejo D., Ortega-Arellano H. (2017). Combination of melatonin and rapamycin for head and neck cancer therapy: Suppression of AKT/mTOR pathway activation, and activation of mitophagy and apoptosis via mitochondrial function regulation. J. Pineal Res..

[130] Naik P.P., Mukhopadhyay S., Praharaj P.P., Bhol C.S., Panigrahi D.P., Mahapatra K.K., Patra S., Saha S., Panda A.K., Panda K. (2020). Secretory clusterin promotes oral cancer cell survival via inhibiting apoptosis by activation of autophagy in AMPK/mTOR/ULK1 dependent pathway. Life Sci..

[131] Shin Y.Y., Seo Y., Oh S., Ahn J., Song M., Kang M., Oh J., Lee D., Kim Y.H., Sung E. (2021). Melatonin and verteporfin synergistically suppress the growth and stemness of head and neck squamous cell carcinoma through the regulation of mitochondrial dynamics. J. Pineal Res..

[132] Nazio F., Bordi M., Cianfanelli V., Locatelli F., Cecconi F. (2019). Autophagy and cancer stem cells: Molecular mechanisms and therapeutic applications. Cell Death Differ..

[133] Praharaj P.P., Panigrahi D.P., Bhol C.S., Patra S., Mishra S.R., Mahapatra K.K., Behera B.P., Singh A., Patil S., Bhutia S.K. (2020). Mitochondrial rewiring through mitophagy and mitochondrial biogenesis in cancer stem cells: A potential target for anti-CSC cancer therapy. Cancer Lett..

[134] Naik P.P., Mukhopadhyay S., Panda P.K., Sinha N., Das C.K., Mishra R., Patil S., Bhutia S.K. (2017). Autophagy regulates cisplatin-induced stemness and chemoresistance via the upregulation of CD 44, ABCB 1 and ADAM 17 in oral squamous cell carcinoma. Cell Prolif..

[135] Shaw J.J., Boyer T.L., Venner E., Beck P.J., Slamowitz T., Caste T., Hickman A., Raymond M.H., Costa-Pinheiro P., Jameson M.J. (2020). Inhibition of Lysosomal Function Mitigates Protective Mitophagy and Augments Ceramide Nanoliposome–Induced Cell Death in Head and Neck Squamous Cell Carcinoma. Mol. Cancer Ther..

[136] Wang J., Gao S., Wang S., Xu Z., Wei L. (2018). Zinc oxide nanoparticles induce toxicity in CAL 27 oral cancer cell lines by activating PINK1/Parkin-mediated mitophagy. Int. J. Nanomed..

[137] Rencelj A., Gvozdenovic N., Cemazar M. (2021). MitomiRs: Their roles in mitochondria and importance in cancer cell metabolism. Radiol. Oncol..

[138] Chen W., Wang P., Lu Y., Jin T., Lei X., Liu M., Zhuang P., Liao J., Lin Z., Li B. (2019). Decreased expression of mitochondrial miR-5787 contributes to chemoresistance by reprogramming glucose metabolism and inhibiting MT-CO3 translation. Theranostics.

[139] Fan S., Tian T., Chen W., Lv X., Lei X., Zhang H., Sun S., Cai L., Pan G., He L. (2019). Mitochondrial miRNA Determines Chemoresistance by Reprogramming Metabolism and Regulating Mitochondrial Transcription. Cancer Res..

[140] Kao Y.-Y., Chou C.-H., Yeh L.-Y., Chen Y.-F., Chang K.-W., Liu C.-J., Chiang C.-Y.F., Lin S.-C. (2019). MicroRNA miR-31 targets SIRT3 to disrupt mitochondrial activity and increase oxidative stress in oral carcinoma. Cancer Lett..

[141] Lo Y.-L., Wang C.-S., Chen Y.-C., Wang T.-Y., Chang Y.-H., Chen C.-J., Yang C.-P. (2020). Mitochondrion-Directed Nanoparticles Loaded with a Natural Compound and a microRNA for Promoting Cancer Cell Death via the Modulation of Tumor Metabolism and Mitochondrial Dynamics. Pharmaceutics.

[142] Cui Z., Bao X., Liu Q., Li Q., Huang L., Wang H., Jiao K. (2019). MicroRNA-378-3p/5p represses proliferation and induces apoptosis of oral squamous carcinoma cells via targeting KLK4. Clin. Exp. Pharmacol. Physiol..

[143] Wu Z., Oeck S., West A.P., Mangalhara K.C., Sainz A.G., Newman L., Zhang X.-O., Wu L., Yan Q., Bosenberg M. (2019). Mitochondrial DNA stress signalling protects the nuclear genome. Nat. Metab..

[144] Lin Y.-H., Lim S.-N., Chen C.-Y., Chi H.-C., Yeh C.-T., Lin W.-R. (2022). Functional Role of Mitochondrial DNA in Cancer Progression. Int. J. Mol. Sci..

[145] Grasso D., Medeiros H., Zampieri L.X., Bol V., Danhier P., Van Gisbergen M.W., Bouzin C., Brusa D., Grégoire V., Smeets H. (2020). Fitter Mitochondria Are Associated With Radioresistance in Human Head and Neck SQD9 Cancer Cells. Front. Pharmacol..

[146] Aminuddin A., Ng P.Y., Leong C.-O., Chua E.W. (2020). Mitochondrial DNA alterations may influence the cisplatin responsiveness of oral squamous cell carcinoma. Sci. Rep..

[147] Wang L., Lv H., Ji P., Zhu X., Yuan H., Jin G., Dai J., Hu Z., Su Y., Ma H. (2018). Mitochondrial DNA copy number is associated with risk of head and neck squamous cell carcinoma in Chinese population. Cancer Med..

[148] Brandon M., Baldi P., Wallace D.C. (2006). Mitochondrial mutations in cancer. Oncogene.

[149] Ju Y.S., Alexandrov L.B., Gerstung M., Martincorena I., Nik-Zainal S., Ramakrishna M., Davies H.R., Papaemmanuil E., Gundem G., Shlien A. (2014). Origins and functional consequences of somatic mitochondrial DNA mutations in human cancer. eLife.

[150] Fendt L., Fazzini F., Weissensteiner H., Bruckmoser E., Schönherr S., Schäfer G., Losso J.L., Streiter G.A., Lamina C., Rasse M. (2020). Profiling of Mitochondrial DNA Heteroplasmy in a Prospective Oral Squamous Cell Carcinoma Study. Cancers.

[151] Bonekamp N.A., Peter B., Hillen H.S., Felser A., Bergbrede T., Choidas A., Horn M., Unger A., Di Lucrezia R., Atanassov I. (2020). Small-molecule inhibitors of human mitochondrial DNA transcription. Nature.

[152] Lai W.-T., Li Y.-J., Wu S.-B., Yang C.-N., Wu T.-S., Wei Y.-H., Deng Y.-T. (2018). Connective tissue growth factor decreases mitochondrial metabolism through ubiquitin-mediated degradation of mitochondrial transcription factor A in oral squamous cell carcinoma. J. Formos. Med. Assoc..

[153] Li W., Zhang L. (2020). Rewiring Mitochondrial Metabolism for CD8+ T Cell Memory Formation and Effective Cancer Immunotherapy. Front. Immunol..

[154] Wu Z.-Z., Wang S., Yang Q.-C., Wang X.-L., Yang L.-L., Liu B., Sun Z.-J. (2020). Increased Expression of SHMT2 Is Associated With Poor Prognosis and Advanced Pathological Grade in Oral Squamous Cell Carcinoma. Front. Oncol..

[155] Liao Y., Wang F., Zhang Y., Cai H., Song F., Hou J. (2021). Silencing SHMT2 inhibits the progression of tongue squamous cell carcinoma through cell cycle regulation. Cancer Cell Int..

[156] Cheng A.N., Cheng L.-C., Kuo C.-L., Lo Y.K., Chou H.-Y., Chen C.-H., Wang Y.-H., Chuang T.-H., Cheng S.-J., Lee A.Y.-L. (2020). Mitochondrial Lon-induced mtDNA leakage contributes to PD-L1–mediated immunoescape via STING-IFN signaling and extracellular vesicles. J. Immunother. Cancer.

[157] Kuo C.-L., Chou H.-Y., Chiu Y.-C., Cheng A.N., Fan C.-C., Chang Y.-N., Chen C.-H., Jiang S.S., Chen N.-J., Lee A.Y.-L. (2020). Mitochondrial oxidative stress by Lon-PYCR1 maintains an immunosuppressive tumor microenvironment that promotes cancer progression and metastasis. Cancer Lett..

[158] Nocquet L., Juin P.P., Souazé F. (2020). Mitochondria at Center of Exchanges between Cancer Cells and Cancer-Associated Fibroblasts during Tumor Progression. Cancers.

[159] Hui S., Ghergurovich J.M., Morscher R.J., Jang C., Teng X., Lu W., Esparza L.A., Reya T., Zhan L., Guo J.Y. (2017). Glucose feeds the TCA cycle via circulating lactate. Nature.

[160] Jiang E., Xu Z., Wang M., Yan T., Huang C., Zhou X., Liu Q., Wang L., Chen Y., Wang H. (2019). Tumoral microvesicle–activated glycometabolic reprogramming in fibroblasts promotes the progression of oral squamous cell carcinoma. FASEB J..

[161] Zhao H., Jiang E., Shang Z. (2021). 3D Co-culture of Cancer-Associated Fibroblast with Oral Cancer Organoids. J. Dent. Res..

[162] Zhang Z., Gao Z., Rajthala S., Sapkota D., Dongre H., Parajuli H., Suliman S., Das R., Li L., Bindoff L.A. (2020). Metabolic reprogramming of normal oral fibroblasts correlated with increased glycolytic metabolism of oral squamous cell carcinoma and precedes their activation into carcinoma associated fibroblasts. Cell Mol. Life Sci..

[163] Saha T., Dash C., Jayabalan R., Khiste S., Kulkarni A., Kurmi K., Mondal J., Majumder P.K., Bardia A., Jang H.L. (2021). Intercellular nanotubes mediate mitochondrial trafficking between cancer and immune cells. Nat. Nanotechnol..

[164] Ding Z., Sigdel K., Yang L., Liu Y., Xuan M., Wang X., Gu Z., Wu J., Xie H. (2020). Nanotechnology-based drug delivery systems for enhanced diagnosis and therapy of oral cancer. J. Mater. Chem. B.

[165] Hossain M., Das U., Dimmock J.R. (2019). Recent advances in α,β-unsaturated carbonyl compounds as mitochondrial toxins. Eur. J. Med. Chem..

[166] Nguyen C., Pandey S. (2019). Exploiting Mitochondrial Vulnerabilities to Trigger Apoptosis Selectively in Cancer Cells. Cancers.

[167] Mani S., Swargiary G., Singh K.K. (2020). Natural Agents Targeting Mitochondria in Cancer. Int. J. Mol. Sci..

[168] Qian Q., Chen W., Cao Y., Cao Q., Cui Y., Li Y., Wu J. (2019). Targeting Reactive Oxygen Species in Cancer via Chinese Herbal Medicine. Oxidative Med. Cell Longev..

[169] Shakeri F., Bianconi V., Pirro M., Sahebkar A. (2020). Effects of Plant and Animal Natural Products on Mitophagy. Oxidative Med. Cell Longev..

[170] Zhu Y., Wen L.-M., Li R., Dong W., Jia S.-Y., Qi M.-C. (2019). Recent advances of nano-drug delivery system in oral squamous cell carcinoma treatment. Eur. Rev. Med. Pharmacol. Sci..

[171] Li H., Zhang Y., Xu M., Yang D. (2022). Current trends of targeted therapy for oral squamous cell carcinoma. J. Cancer Res. Clin. Oncol..

[172] Cryer A.M., Thorley A.J. (2019). Nanotechnology in the diagnosis and treatment of lung cancer. Pharmacol. Ther..

[173] Bayda S., Adeel M., Tuccinardi T., Cordani M., Rizzolio F. (2020). The History of Nanoscience and Nanotechnology: From Chemical–Physical Applications to Nanomedicine. Molecules.

[174] Zhang Y., Li M., Gao X., Chen Y., Liu T. (2019). Nanotechnology in cancer diagnosis: Progress, challenges and opportunities. J. Hematol. Oncol..

[175] Şen Ö., Emanet M., Ciofani G. (2021). Nanotechnology-Based Strategies to Evaluate and Counteract Cancer Metastasis and Neoangiogenesis. Adv. Health Mater..

[176] Wang Y., Zhang W., Sun P., Cai Y., Xu W., Fan Q., Hu Q., Han W. (2019). A Novel Multimodal NIR-II Nanoprobe for the Detection of Metastatic Lymph Nodes and Targeting Chemo-Photothermal Therapy in Oral Squamous Cell Carcinoma. Theranostics.

[177] Li R., Gao R., Wang Y., Liu Z., Xu H., Duan A., Zhang F., Ma L. (2020). Gastrin releasing peptide receptor targeted nano-graphene oxide for near-infrared fluorescence imaging of oral squamous cell carcinoma. Sci. Rep..

[178] Allemailem K.S., Almatroudi A., Alsahli M.A., Aljaghwani A., El-Kady A.M., Rahmani A.H., Khan A.A. (2021). Novel Strategies for Disrupting Cancer-Cell Functions with Mitochondria-Targeted Antitumor Drug–Loaded Nanoformulations. Int. J. Nanomed..

[179] Afrasiabi M., Seydi E., Rahimi S., Tahmasebi G., Jahanbani J., Pourahmad J. (2021). The selective toxicity of superparamagnetic iron oxide nanoparticles (SPIONs) on oral squamous cell carcinoma (OSCC) by targeting their mitochondria. J. Biochem. Mol. Toxicol..

[180] Jahanbani J., Ghotbi M., Shahsavari F., Seydi E., Rahimi S., Pourahmad J. (2020). Selective anticancer activity of superparamagnetic iron oxide nanoparticles (SPIONs) against oral tongue cancer using in vitro methods: The key role of oxidative stress on cancerous mitochondria. J. Biochem. Mol. Toxicol..

[181] Wang S.-W., Lee C.-H., Lin M.-S., Chi C.-W., Chen Y.-J., Wang G.-S., Liao K.-W., Chiu L.-P., Wu S.-H., Huang D.-M. (2020). ZnO Nanoparticles Induced Caspase-Dependent Apoptosis in Gingival Squamous Cell Carcinoma through Mitochondrial Dysfunction and p70S6K Signaling Pathway. Int. J. Mol. Sci..

[182] Satapathy S.R., Nayak A., Siddharth S., Das S., Nayak D., Kundu C.N. (2018). Metallic gold and bioactive quinacrine hybrid nanoparticles inhibit oral cancer stem cell and angiogenesis by deregulating inflammatory cytokines in p53 dependent manner. Nanomed. Nanotechnol. Biol. Med..

[183] Hu T., Qin Z., Shen C., Gong H.-L., He Z.-Y. (2021). Multifunctional Mitochondria-Targeting Nanosystems for Enhanced Anticancer Efficacy. Front. Bioeng. Biotechnol..

[184] Lai K.-C., Chueh F.-S., Hsiao Y.-T., Cheng Z.-Y., Lien J.-C., Liu K.-C., Peng S.-F., Chung J.-G. (2019). Gefitinib and curcumin-loaded nanoparticles enhance cell apoptosis in human oral cancer SAS cells in vitro and inhibit SAS cell xenografted tumor in vivo. Toxicol. Appl. Pharmacol..

[185] Mariadoss A.V.A., Vinayagam R., Senthilkumar V., Paulpandi M., Murugan K., Xu B., Gothandam K.M., Kotakadi V.S., David E. (2019). Phloretin loaded chitosan nanoparticles augments the pH-dependent mitochondrial-mediated intrinsic apoptosis in human oral cancer cells. Int. J. Biol. Macromol..

[186] Fan H.-Y., Zhu Z.-L., Zhang W.-L., Yin Y.-J., Tang Y.-L., Liang X.-H., Zhang L. (2020). Light stimulus responsive nanomedicine in the treatment of oral squamous cell carcinoma. Eur. J. Med. Chem..

[187] Wang X., Li S., Liu H. (2021). Co-delivery of chitosan nanoparticles of 5-aminolevulinic acid and shGBAS for improving photodynamic therapy efficacy in oral squamous cell carcinomas. Photodiagnosis Photodyn. Ther..

[188] Dash S.R., Chatterjee S., Sinha S., Das B., Paul S., Pradhan R., Sethy C., Panda R., Tripathy J., Kundu C.N. (2021). NIR irradiation enhances the apoptotic potentiality of quinacrine-gold hybrid nanoparticles by modulation of HSP-70 in oral cancer stem cells. Nanomed. Nanotechnol. Biol. Med..

[189] Mendes B.B., Sousa D.P., Conniot J., Conde J. (2021). Nanomedicine-based strategies to target and modulate the tumor microenvironment. Trends Cancer.

[190] Shi J., Kantoff P.W., Wooster R., Farokhzad O.C. (2017). Cancer nanomedicine: Progress, challenges and opportunities. Nat. Rev. Cancer.

[191] Gong X., Tang H., Yang K. (2021). PER1 suppresses glycolysis and cell proliferation in oral squamous cell carcinoma via the PER1/RACK1/PI3K signaling complex. Cell Death Dis..

[192] Pai S., Yadav V.K., Kuo K.-T., Pikatan N.W., Lin C.-S., Chien M.-H., Lee W.-H., Hsiao M., Chiu S.-C., Yeh C.-T. (2021). PDK1 Inhibitor BX795 Improves Cisplatin and Radio-Efficacy in Oral Squamous Cell Carcinoma by Downregulating the PDK1/CD47/Akt-Mediated Glycolysis Signaling Pathway. Int. J. Mol. Sci..

[193] Martínez-Reyes I., Chandel N.S. (2020). Mitochondrial TCA cycle metabolites control physiology and disease. Nat. Commun..

[194] Sharma N., Pasala M.S., Prakash A. (2019). Mitochondrial DNA: Epigenetics and environment. Environ. Mol. Mutagen..

[195] Ghosh-Choudhary S.K., Liu J., Finkel T. (2021). The role of mitochondria in cellular senescence. FASEB J..

[196] Andrieux P., Chevillard C., Cunha-Neto E., Nunes J.P.S. (2021). Mitochondria as a Cellular Hub in Infection and Inflammation. Int. J. Mol. Sci..

[197] Greten F.R., Grivennikov S.I. (2019). Inflammation and Cancer: Triggers, Mechanisms, and Consequences. Immunity.

[198] Sant’Ana M.S.P., de Cáceres C.V.B.L., Lima L.A., Soares C.D., Radhakrishnan R., Gomez R.S., Vargas P.A., Fonseca F.P. (2022). Expression of mitochondrial dynamic markers in adenoid cystic carcinoma. J. Oral Pathol. Med..

[199] Qiu L., Liu Z., Wu C., Chen W., Chen Y., Zhang B., Li J., Liu H., Huang N., Jiang Z. (2020). C6-ceramide induces salivary adenoid cystic carcinoma cell apoptosis via IP3R-activated UPR and UPR-independent pathways. Biochem. Biophys. Res. Commun..

